# Supplementary material for: What’s in a Meow? A Study on Human Classification and Interpretation of Domestic Cat Vocalizations
Source: Animals (Basel). 2020 Dec 14;10(12):2390. doi: 10.3390/ani10122390 (PMC7765146; doi:10.3390/ani10122390)
Supplement: Supplementary file 1 [file animals-10-02390-s001.pdf]

What's in a "meow": investigating cat–human communication

Informazioni generali:

1. **Genere:**

   ☐ Femmina ☐ Maschio

2. **Età:**…………..

3. **Stato civile**
   1. Coniugata/o
   2. Divorziata/o – Separata/o
   3. Non sposata/o
   4. Vedova/o

4. **Ha dei figli:** ☐ Sì ☐ No

5. **Grado di istruzione raggiunto:**
   1. Diploma scuola media superiore ☐ Liceo ☐ Istituto tecnico ☐ Istituto professionale
   2. Laurea in …………………
   3. Formazione Post-Universitaria: …………………

6. **Professione/attività di lavoro:** ……………………………

7. **E' cresciuto con animali domestici in casa?** ☐ Sì ☐ No

7a. Se <u>Sì</u>, che tipo di animali domestici?……………………………..…………….

8. **Attualmente ha animali domestici in casa?** ☐ Sì ☐ No

8a. Se <u>Sì</u>, che tipo di animali domestici?…………………………………………….

Esperienza e relazione con i gatti:

Le domande riportate di seguito Le permettono di descrivere il Suo rapporto con i gatti:

	PER NULLA/POCA	DISCRETA	MOLTA	MOLTISSIMA
1. Ha esperienza di vita insieme a gatti?				

	PER NULLA/POCA	ABBASTANZA	SPESSO	SEMPRE
2. Quanto spesso interagisce con i gatti?				

	PER NULLA/POCA	DISCRETA	MOLTA	MOLTISSIMA
3. Quanta affinità prova per i gatti?				

AES (Paul, 2000)

1	2	3	4	5	6	7	8	9
**Molto in disaccordo**	**In disaccordo**	**Moderatamente in disaccordo**	**Leggermente in disaccordo**	**Indeciso**	**Leggermente d’accordo**	**Moderatamente d’accordo**	**D’accordo**	**Molto d’accordo**

1. Non penso che agli animali dello zoo, se sono ben gestiti e ben nutriti, dispiaccia essere tenuti in gabbia.	1 – 2 – 3 – 4 – 5 – 6 – 7 – 8 – 9
2. Spesso i gatti miagolano e ci infastidiscono per ottenere cibo senza essere davvero affamati.	1 – 2 – 3 – 4 – 5 – 6 – 7 – 8 – 9
3. Mi dispiace vedere, nei programmi televisivi sulla fauna selvatica, animali inseguiti e uccisi dai leoni.	1 – 2 – 3 – 4 – 5 – 6 – 7 – 8 – 9
4. I cani che ululano e abbaiano quando vengono lasciati da soli mi infastidiscono.	1 – 2 – 3 – 4 – 5 – 6 – 7 – 8 – 9
5. I film tristi sugli animali mi lasciano spesso con un groppo in gola.	1 – 2 – 3 – 4 – 5 – 6 – 7 – 8 – 9
6. Gli animali meritano di essere rimproverati quando non si comportano bene.	1 – 2 – 3 – 4 – 5 – 6 – 7 – 8 – 9
7. Vedere un animale da solo in gabbia mi rende triste.	1 – 2 – 3 – 4 – 5 – 6 – 7 – 8 – 9
8. Le persone che coccolano e baciano i loro animali domestici in pubblico mi infastidiscono.	1 – 2 – 3 – 4 – 5 – 6 – 7 – 8 – 9
9. Un gatto amichevole che fa le fusa di solito mi mette allegria.	1 – 2 – 3 – 4 – 5 – 6 – 7 – 8 – 9
10. Mi dispiace vedere animali anziani e vulnerabili.	1 – 2 – 3 – 4 – 5 – 6 – 7 – 8 – 9
11. A volte i cani guaiscono e piagnucolano senza un reale motivo.	1 – 2 – 3 – 4 – 5 – 6 – 7 – 8 – 9
12. Molte persone sono eccessivamente affezionate ai loro animali domestici.	1 – 2 – 3 – 4 – 5 – 6 – 7 – 8 – 9
13. Mi arrabbio molto quando vedo maltrattare gli animali.	1 – 2 – 3 – 4 – 5 – 6 – 7 – 8 – 9
14. Affezionarsi troppo ad un animale domestico è sciocco.	1 – 2 – 3 – 4 – 5 – 6 – 7 – 8 – 9

15. Gli animali domestici hanno un’influenza molto positiva sul mio umore.	1 – 2 – 3 – 4 – 5 – 6 – 7 – 8 – 9
16. Talvolta mi stupisce vedere quanto alcune persone rimangano sconvolte dalla morte del proprio animale domestico.	1 – 2 – 3 – 4 – 5 – 6 – 7 – 8 – 9
17. Mi fa piacere dare da mangiare agli uccelli briciole di cibo.	1 – 2 – 3 – 4 – 5 – 6 – 7 – 8 – 9
18. Mi addolora vedere animali sofferenti.	1 – 2 – 3 – 4 – 5 – 6 – 7 – 8 – 9
19. Spesso le persone danno troppa importanza a sentimenti e sensibilità degli animali.	1 – 2 – 3 – 4 – 5 – 6 – 7 – 8 – 9
20. E’ irritante quando i cani tentano di accogliermi saltandomi addosso e leccandomi.	1 – 2 – 3 – 4 – 5 – 6 – 7 – 8 – 9
21. Se vedessi un cane o un cucciolo che sembra essersi smarrito cercherei sempre di aiutarlo.	1 – 2 – 3 – 4 – 5 – 6 – 7 – 8 – 9
22. Odio vedere gli uccelli chiusi in gabbie in cui non c’è alcuno spazio per svolazzare.	1 – 2 – 3 – 4 – 5 – 6 – 7 – 8 – 9
23. Trovo difficile vedere le situazioni dal punto di vista di un gatto.	1 – 2 – 3 – 4 – 5 – 6 – 7 – 8 – 9
24. Capisco con facilità se un gatto vuole comunicare con me.	1 – 2 – 3 – 4 – 5 – 6 – 7 – 8 – 9
25. Riesco a capire rapidamente e intuitivamente come si sente un gatto.	1 – 2 – 3 – 4 – 5 – 6 – 7 – 8 – 9
26. Sono bravo a predire quello che un gatto farà.	1 – 2 – 3 – 4 – 5 – 6 – 7 – 8 – 9

La ringraziamo per la collaborazione!
